# Widely targeted metabolomics of different tissues in *Rubus corchorifolius*

**DOI:** 10.1515/biol-2022-0996

**Published:** 2025-03-07

**Authors:** Xiangmei Chen, Liang Liang, Shan Chang, Xiang Chun, Yun Qing

**Affiliations:** Inner Mongolia Minzu University, Tongliao, China; Key Laboratory of Mongolian Medicine Research and Development Engineering Ministry of Education, No. 996 West Ramulun Street (West), Horqin District, Tongliao, Inner Mongolia Autonomous Region, 028000, China

**Keywords:** medicinal plant, metabolic profiles, metabolomics, *Rubus corchorifolius*, tissue-specific metabolism

## Abstract

*Rubus corchorifolius*, a medicinal plant of the *Rosaceae* family, is known for its diverse bioactive compounds. This study employs widely targeted metabolomics to investigate the metabolic profiles of leaf, stem, and flower tissue from *R. corchorifolius*. Using ultra-performance liquid chromatography coupled with tandem mass spectrometry, we identified 1,946 metabolites across the three tissue types. Multivariate statistical analyses revealed distinct metabolic signatures for each tissue, with flowers showing the most distinctive profile. Differential accumulation of flavonoids, phenolic acids, and primary metabolites reflected the specialised functions of each tissue type. Pathway enrichment analysis highlighted tissue-specific metabolic activities, including flavonoid biosynthesis in flowers and chlorophyll metabolism in leaves. This comprehensive metabolic characterisation provides a foundation for further investigations into the biosynthetic pathways and physiological functions of bioactive compounds in *R. corchorifolius*, potentially guiding future applications in medicine and agriculture.

## 1 Introduction

Natural products have long been recognised for their significant therapeutic potential in promoting human health. Recent studies have demonstrated the diverse applications of compounds derived from both plant and animal sources in preventing and treating various ailments. For instance, camel whey protein hydrolysates have shown promising effects in managing colorectal cancer by inducing G2/M cell cycle arrest [1]. Plant-based bioactive compounds, such as safranal from saffron, have exhibited hepatoprotective properties, offering potential strategies for addressing liver cancer through inhibition of oxidative stress and alleviation of inflammation [2]. Additionally, natural products have demonstrated efficacy in various health applications. *Rhizoma polygonati* compounds have shown potential in treating COVID-19, highlighting their possible role in antiviral therapy [3]. The combination of Hibiscus extract with cisplatin has demonstrated reduced liver toxicity while increasing effectiveness against lung cancer cells, suggesting a novel approach in cancer treatment [4]. Furthermore, the combination of crocin (a compound from saffron) with sorafenib has shown improved tumour-inhibiting effects in a rat model of cirrhotic hepatocellular carcinoma, indicating potential synergistic effects in cancer therapy [5].

Widely targeted metabolomics usually employs ultra-performance liquid chromatography (UPLC) coupled with tandem mass spectrometry and a pre-defined metabolite database for metabolite identification and quantification [6]. Compared with the traditional untargeted approach, widely targeted metabolomics greatly improves the efficiency and reliability of metabolite annotation. Meanwhile, it expands the metabolite coverage compared with targeted methods, which only focus on a limited number of known compounds [7]. Widely targeted metabolomics has been successfully applied to study the metabolic variations in various plant species and response to different biotic and abiotic factors [8,9,10].

*Rubus corchorifolius*, commonly known as library hook vine, is a perennial climbing shrub of the genus *Rubus* in the rose family (*Rosaceae*) [11]. It is native to East Asia and Southeast Asia, with a distribution spanning from Japan and Korea to China and Vietnam [12]. In China, *R. corchorifolius* is mainly found in the southern regions, including Zhejiang, Jiangxi, Fujian and Taiwan provinces [13]. *R. corchorifolius* is shrub with slender, prickly stems, simple ovate to lanceolate leaves, small white flowers, and aggregate red fruits. The fruits of *R. corchorifolius*, which turn red upon ripening, are edible and have a unique flavour. The leaves and roots of this plant are used as a folk medicine for treating various ailments, such as traumatic injury, rheumatoid arthritis and irregular menstruation [14]. Previous phytochemical studies have identified multiple bioactive constituents from *R. corchorifolius*, including triterpenes, flavonoids, tannins and steroids [15,16]. However, the overall metabolic profiles of different tissues in *R. corchorifolius* remain largely unexplored.

This study aims to address this knowledge gap by employing widely targeted metabolomics to investigate the tissue-specific metabolic profiles of *R. corchorifolius*. This work is essential because it will provide a comprehensive understanding of the metabolic composition across different plant tissues. The findings will have a significant impact on Rubus improvement by identifying metabolic markers associated with desirable traits. Moreover, these metabolomic studies will help enhance the content of desirable chemicals in this plant by elucidating biosynthetic pathways. While Rubus is not the sole natural source for any specific metabolite, it is known for its unique combination of bioactive compounds, particularly anthocyanins and ellagitannins. Our objectives are the following: (a) characterise the metabolic differences among leaf, stem and flower tissues; (b) identify key metabolites and pathways that contribute to the plant’s medicinal properties; and (c) provide a foundation for future studies on metabolic engineering and quality improvement in *R. corchorifolius*.

## 2 Materials and methods

### 2.1 Plant material and sample preparation

*Rubus corchorifolius* plants were collected from the natural habitats in Zhejiang Province, China, in May 2023. The mature leaves, stems and flowers were separately harvested, immediately frozen in liquid nitrogen and stored at −80°C until metabolite extraction.

For each tissue type, three biological replicates were prepared. The samples were ground into fine powder in liquid nitrogen using a mortar and pestle. Approximately 100 mg of powder was weighed and extracted with 1.2 ml pre-cooled 70% methanol (containing 0.1% formic acid) by vortexing for 30 s. The mixture was sonicated at 4°C for 10 min and then placed at −20°C for 1 h. After centrifugation at 12,000*g* and 4°C for 15 min, the supernatant was collected and filtered through a 0.22 μm membrane for liquid chromatography–mass spectrometry analysis.

Voucher specimens (XGZM230101, XGZM230102 and XGZM230103) have been deposited in the herbarium of Mongolian Medical College of Inner Mongolia Minzu University.

### 2.2 Widely targeted metabolomics

Metabolic profiling was performed on an ExionLC™ AD UPLC system coupled with a SCIEX Triple TOF 5600+ mass spectrometer (MS). Chromatographic separation was carried out on an Acquity UPLC BEH C18 column (100 mm × 2.1 mm, 1.7 μm). The mobile phases consisted of (A) water containing 0.1% formic acid and (B) acetonitrile containing 0.1% formic acid. The elution gradient was set as follows: 0–1 min, 5% B; 1–9 min, 5–95% B; 9–10 min, 95% B; 10–10.1 min, 95–5% B; and 10.1–14 min, 5% B. The flow rate was 0.4 ml/min and the injection volume was 2 μl.

The MS was operated in both positive and negative ion modes. The electrospray ionisation source conditions were as follows: ion source gas 1, 60 psi; ion source gas 2, 60 psi; curtain gas, 30 psi; source temperature, 600°C; ion spray voltage floating, 5,500 V in positive mode and −4,500 V in the negative mode. The TOF MS scan range was 60–1,000 Da and the product ion scan range was 25–1,000 Da. The declustering potential and collision energy were optimised for individual metabolites.

### 2.3 Data processing and metabolite identification

The raw MS data were converted to mzXML files using the ProteoWizard MSConvert tool and processed with an in-house program, which was developed using R and based on XCMS for peak detection, extraction, alignment and integration. The metabolites were identified by matching the accurate mass-to-charge ratio values and MS/MS fragmentation patterns with a pre-constructed metabolite database, which contains information on >800 metabolite standards. The mass error threshold was set to 10 ppm and the MS/MS spectral similarity threshold was set to 0.8.

### 2.4 Statistical analysis

The processed metabolite data were normalised by the total peak intensity and subjected to log transformation and Pareto scaling before statistical analysis. The overall metabolic differences between tissue types were visualised using principal component analysis (PCA) and orthogonal partial least-squares discriminant analysis (OPLS-DA) in SIMCA software (version 14.1). The quality of the OPLS-DA model was assessed by the parameters R2X, R2Y, and Q2 and validated by a permutation test (*n* = 200).

The differential metabolites between tissue comparisons were determined by the combination of variable importance in projection (VIP) >1 from the OPLS-DA model and fold change (FC) >2 or <0.5. The hierarchical cluster analysis was performed using the “pheatmap” package in R to reveal the accumulation patterns of differential metabolites across samples.

Kyoto Encyclopedia of Genes and Genomes (KEGG) pathway enrichment analysis was conducted to explore the metabolic pathways that were significantly altered between tissues. The *p*-values were adjusted for multiple testing using the Benjamin–Hochberg method. Pathways with adjusted *p*-values of <0.05 were considered as significantly enriched.

## 3 Results

### 3.1 Overview of metabolite profiles in different tissues of *R. corchorifolius*

**Figure 1:**
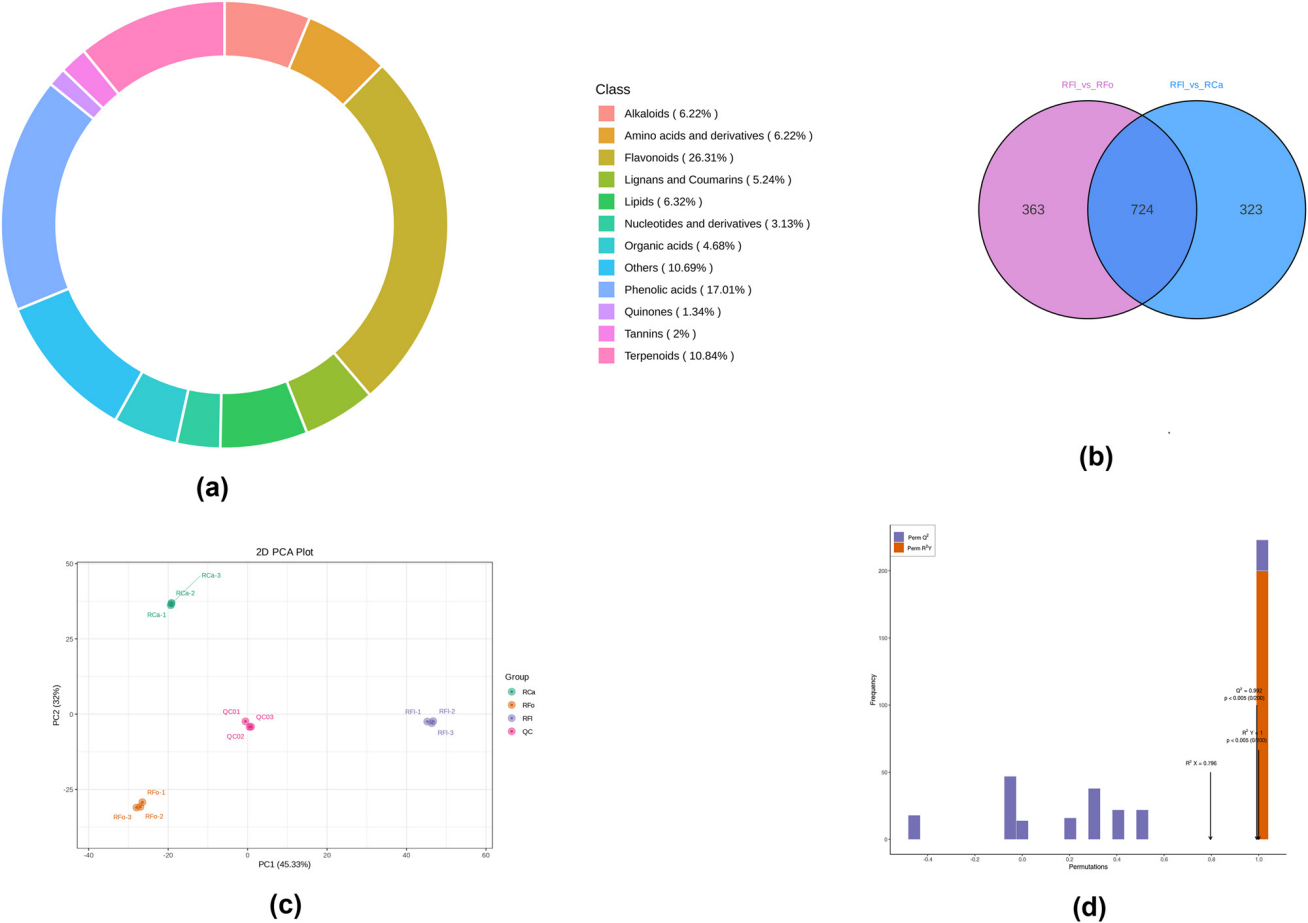
Metabolite profiling and analysis of different tissues in *R. corchorifolius*. (a) Distribution of main chemical classes among the detected metabolites. The pie chart illustrates the relative proportions of different metabolite classes, with flavonoids (26.31%) and phenolic acids (17.01%) being the most abundant. (b) Venn diagram showing the number of common and unique metabolites between RFl_vs_RFo (flower vs flower bud) and RFl_vs_RCa (flower vs calyx) comparisons; 724 metabolites are shared between the two comparisons. (c) PCA score plot of metabolite profiles in different tissues. Each point represents a sample, with colours indicating tissue types: RCa (green, calyx), RFo (orange, flower bud), RFl (purple, flower), and QC (pink, quality control). (d) OPLS-DA model validation by the permutation test. The graph shows the R2 and Q2 values for the original model (far right) and permuted models, indicating the model’s robustness and predictive power.

Using the widely targeted metabolomics approach, 1,946 metabolites were identified in the leaf, stem and flower tissues of *R. corchorifolius*. These metabolites belonged to diverse chemical classes, mainly including flavonoids, phenolic acids, amino acids and derivatives, lipids and organic acids (Figure 1a). The Venn diagram shows that 1,408 metabolites were commonly detected in all three tissues, while 363, 323 and 724 metabolites were uniquely identified in the leaf, stem and flower samples, respectively (Figure 1b).

The PCA score plot reveals a clear separation between the three tissue types (Figure 1c). The first two principal components explained 64.8% of the total variance, with PC1 (45.3%) representing the difference between the flower and the other two tissues and PC2 (32%) representing the difference between the leaf and stem. The OPLS-DA model further demonstrated the distinctive metabolic phenotypes of different tissues (Figure 1d). The model parameters (R2X = 0.796, R2Y = 1, and Q2 = 0.992) indicated good fitness and predictability. No overfitting was observed in the permutation test.

### 3.2 Differential metabolites between tissues

To investigate the metabolic differences between tissues, pairwise comparisons were performed using the criteria of VIP >1 and FC >2 or <0.5. A total of 1,047, 1,087 and 947 metabolites were identified as differentially accumulated in flower vs stem, flower vs leaf and leaf vs stem comparisons, respectively. Interestingly, the number of upregulated metabolites was much higher than downregulated ones in both flower vs stem (790 vs 297) and flower vs leaf (773 vs 274) comparisons, implying the highly active metabolic status in flower tissue. In contrast, the leaf vs stem comparison showed a relatively balanced number of upregulated (493) and downregulated (454) metabolites.

**Figure 2:**
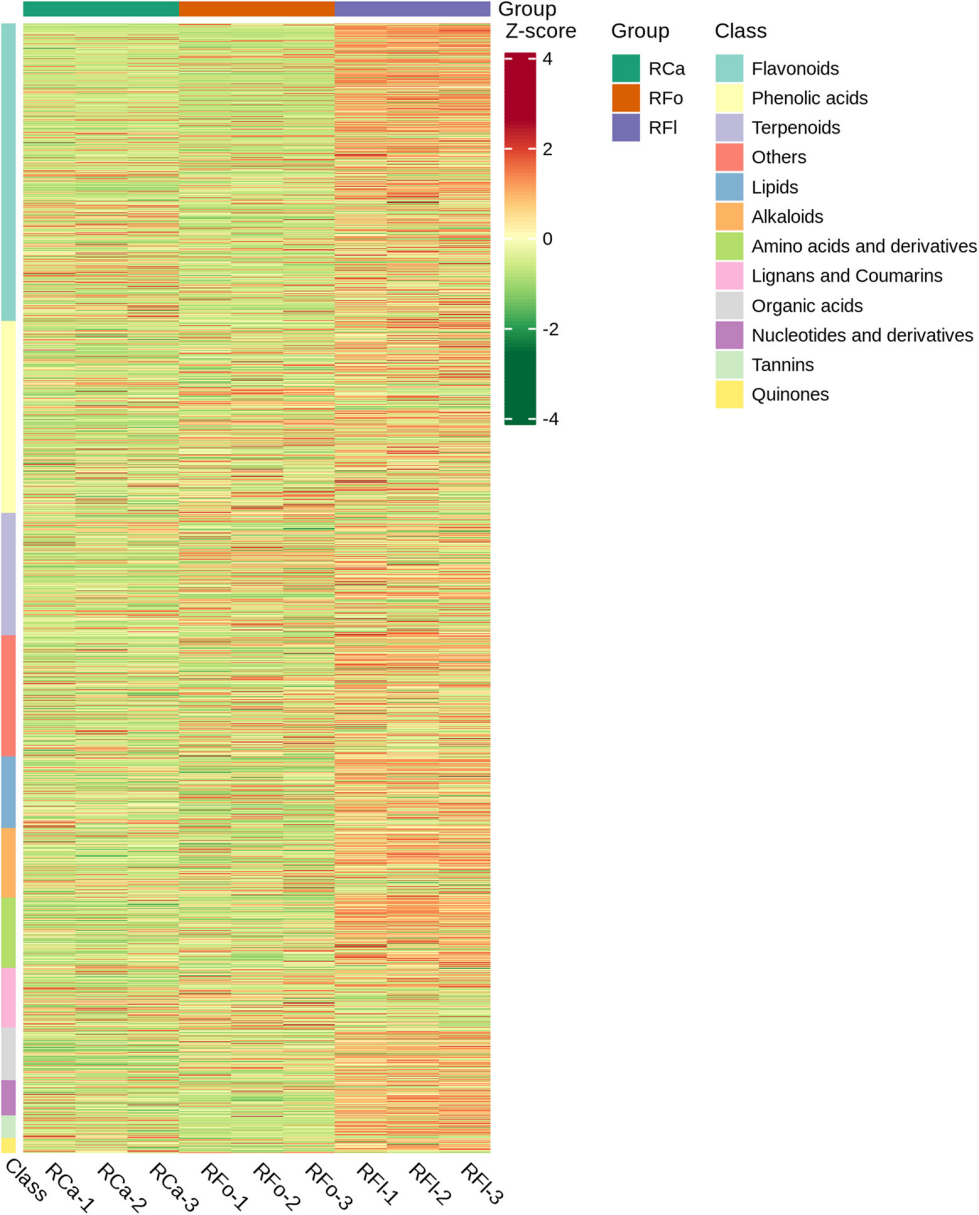
Hierarchical clustering heatmap of differential metabolites across tissues of *R. corchorifolius*. The heatmap displays the relative abundance of differential metabolites (rows) in different tissue samples (columns). The colour scale represents the *Z*-score normalised abundance, with red indicating high abundance and green indicating low abundance. Tissue types are labelled at the top: RCa (calyx), RFo (flower bud) and RFl (flower). The right-side bar shows the metabolite classes, revealing distinct accumulation patterns across tissues.

The clustering heatmap displays the distinct accumulation patterns of differential metabolites across tissues (Figure 2). The flower samples were clearly separated from leaf and stem samples, and many metabolites showed higher abundance in flowers. The most abundant metabolites in flowers include several flavonoids, such as kaempferol and quercetin glycosides, which are important pigments for flower colouration and also possess antioxidant and ultraviolet (UV)-protective functions [17]. The leaf samples contained relatively higher levels of chlorophyll-related compounds, such as pheophorbide *a* and hydroxychlorophyll *a*, consistent with the primary role of leaves in photosynthesis. In addition, some phenolic acids (e.g. coumaric acid and caffeic acid derivatives) were enriched in leaves, which may be involved in defence against herbivores and pathogens [18]. The stem samples accumulated more organic acids and amino acids, including citric acid, malic acid and aspartic acid, which are important components for energy metabolism and nutrient transport [19].

### 3.3 KEGG pathway analysis

**Figure 3:**
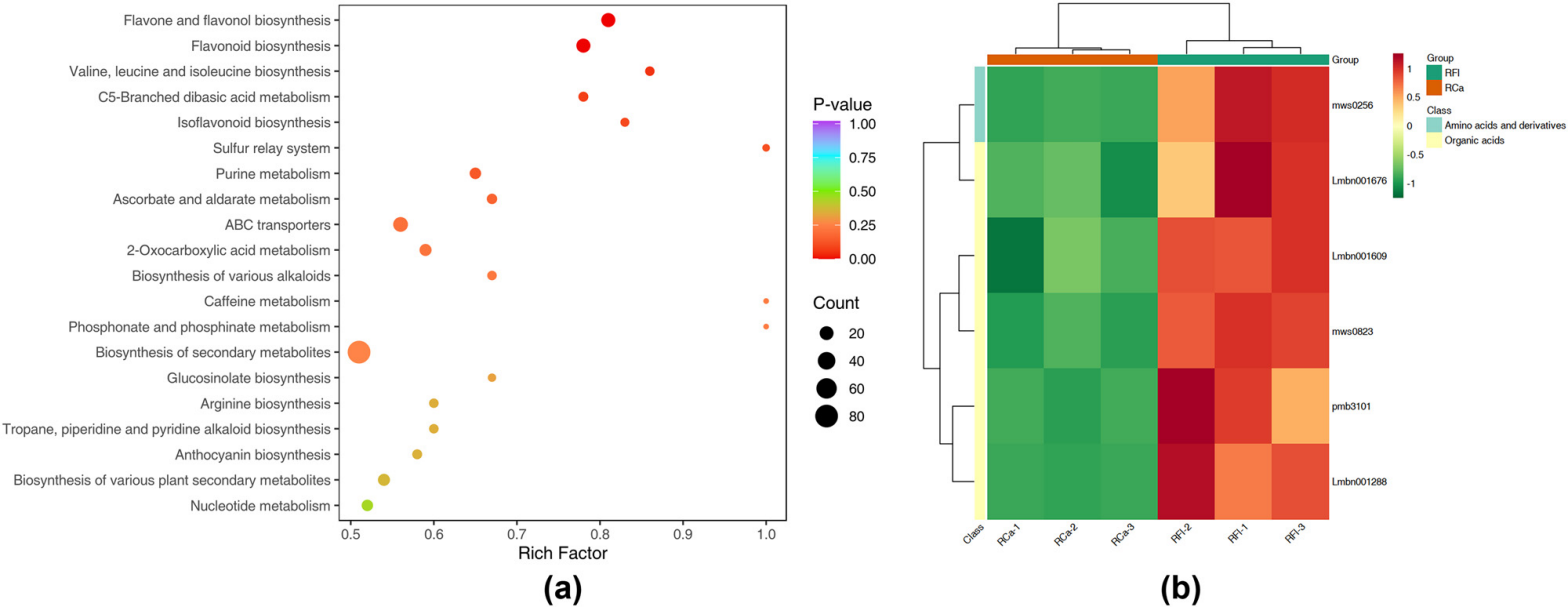
KEGG pathway enrichment analysis of differential metabolites between tissues of *R. corchorifolius*. (a) Bubble plot showing the top 20 enriched KEGG pathways. The *x*-axis represents the rich factor (ratio of differential metabolites to total metabolites in a pathway), and the *y*-axis lists the pathway names. Bubble size indicates the count of differential metabolites, while the colour represents the *p*-value (red: more significant, blue: less significant). (b) Heatmap of key differential metabolites involved in enriched pathways. Rows represent individual metabolites, and columns represent different tissue samples (RFl: flower, RCa: calyx). The colour scale indicates the relative abundance of metabolites (red: high, green: low).

To further understand the metabolic pathways involved in the tissue-specific accumulation of metabolites, KEGG enrichment analysis was performed. A total of 92, 89 and 93 pathways were significantly enriched (adjusted *p* < 0.05) for the differential metabolites in flower vs stem, flower vs leaf and leaf vs stem comparisons, respectively. The top enriched pathways are shown in Figure 3a.

In the flower vs stem comparison, “flavonoid biosynthesis,” “flavone and flavonol biosynthesis” and “anthocyanin biosynthesis” were among the most significantly enriched pathways, which is in line with the abundant accumulation of flavonoids in flowers. The enrichment of the “phenylpropanoid biosynthesis” pathway was also observed, indicating the active biosynthesis of phenolic compounds. In addition, the “ABC transporters” pathway was highly enriched, suggesting the important roles of transporters in the distribution of metabolites between flowers and stems.

In the flower vs leaf comparison, besides the flavonoid-related pathways, “carotenoid biosynthesis” was significantly enriched. Carotenoids are a class of terpenoid pigments that contribute to the yellow, orange and red colours of the flowers [20]. The “cutin, suberin and wax biosynthesis” pathway was also enriched, consistent with the protective functions of cuticle and wax layers in aerial plant tissues.

For the leaf vs stem comparison, the most significantly enriched pathway was “porphyrin and chlorophyll metabolism,” reflecting the crucial roles of leaves in photosynthetic light harvesting. The enrichment of the “phenylpropanoid biosynthesis,” “flavonoid biosynthesis” and “stilbenoid, diarylheptanoid and gingerol biosynthesis” pathways indicated the accumulation of diverse phenolic compounds in leaves, which may be related to defence mechanisms. Moreover, several amino acid biosynthetic pathways, such as “phenylalanine, tyrosine and tryptophan biosynthesis” and “cysteine and methionine metabolism,” were also enriched, implying the active amino acid metabolism in leaves (Figure 3b).

## 4 Discussion

In this study, we applied a widely targeted metabolomics approach to investigate the metabolic profiles of different tissues (leaf, stem and flower) in *R. corchorifolius*. Our results revealed significant metabolic variations among tissues, with flowers exhibiting the most distinctive metabolite composition compared with leaves and stems.

The detection of 1,946 metabolites showcases the comprehensive coverage of widely targeted metabolomics in characterising plant metabolomes. Commonly identified metabolites provide a general metabolic background for *R. corchorifolius*, while tissue-specific metabolites indicate specialised functions of different organs.

Flavonoids accumulate abundantly in *R. corchorifolius* flowers, consistent with their role in flower colouration and pollinator attraction [21]. These compounds also protect reproductive tissues from oxidative damage and UV radiation [22]. The enrichment of flavonoid-related pathways in flowers supports their active biosynthesis.

Leaf tissues accumulated chlorophyll-related compounds and phenolic acids, essential for photosynthesis and defence mechanisms [23]. The stem tissue showed a high abundance of organic acids and amino acids, which are the key components in the central carbon and nitrogen metabolism [24]. This active primary metabolism in stems likely relates to their role in inter-organ connection and nutrient transport.

Our findings on the tissue-specific accumulation of metabolites in *R. corchorifolius* align with recent studies on metabolic profiling in other plant species. For instance, Nurtay et al. [25] demonstrated that metabolite distribution in different plant organs of *Rhizoma polygonati* from Mount Tai is closely related to their physiological functions and potential medicinal applications. The differential accumulation of bioactive compounds observed in our study is consistent with the findings of Nurtay et al. [25], who reported on the unique metabolic profile of Mount Tai-Rhizoma Polygonati and its potential applications in addressing chronic and hidden hunger, as well as its possible use as an anti-COVID-19 agent. This approach is further supported by Ahmad et al. [26] in their computational study on the androgen receptor as a potential anti-infective therapy target for COVID-19. Their study demonstrates the importance of understanding specific molecular interactions and motifs in natural compounds, which can be applied to the study of plant metabolites and their potential therapeutic applications. Additionally, the recent research by Ahmad et al. [27] on the microevolution and phylogenomic characterisation of respiratory syncytial virus highlights the significance of comprehensive mutational analysis across entire genomes. This approach could be adapted to plant metabolomics, providing more detailed insights into the genetic basis of metabolite production and accumulation in different plant tissues, thereby enhancing our understanding of the medicinal properties of plants such as *R. corchorifolius*.

It is worth noting that this study only focused on the metabolic profiles at one developmental stage of *R. corchorifolius*. The metabolite composition may exhibit dynamic changes during different growth and developmental stages, as well as under various environmental conditions [28]. Further studies are needed to investigate the temporal and spatial variations of metabolites in *R. corchorifolius* and to elucidate the underlying regulatory mechanisms.

Although this study provides a comprehensive metabolic profile of *R. corchorifolius* tissues, we recognise the need for further investigation into the biosynthetic pathways of the identified metabolites. Future studies should employ integrated omics approaches, combining metabolomics with transcriptomics and proteomics, to elucidate the enzymatic pathways and regulatory mechanisms underlying the production of key metabolites.

## 5 Conclusion

This study provides a comprehensive metabolic characterisation of leaf, stem and flower tissues in *R. corchorifolius* using widely targeted metabolomics. The distinct metabolite profiles and differentially accumulated metabolites among tissues reflect their specialised metabolic functions. The flavonoid-rich metabolite profile of flowers is associated with their ecological roles in attracting pollinators and protecting reproductive organs. The accumulation of chlorophyll-related compounds and phenolic acids in leaves is related to photosynthesis and defence responses. The stems are characterised by a relatively high abundance of primary metabolites involved in central metabolism. By identifying tissue-specific metabolite accumulation patterns, our study offers insights into the spatial regulation of secondary metabolism in this medicinal plant. This knowledge can guide future metabolic engineering efforts to enhance the production of desirable compounds, such as specific flavonoids or phenolic acids, in targeted tissues. Moreover, the identification of metabolic markers associated with different plant parts can contribute to the development of quality control measures for *R. corchorifolius*-derived products, ensuring consistency in their medicinal properties. Future studies building on these findings could focus on elucidating the regulatory mechanisms controlling the biosynthesis of key metabolites, potentially leading to strategies for optimising the nutritional and medicinal value of *R. corchorifolius* fruits and other plant parts.
